# Type 2 diabetes is associated with increased risk of dementia, but not mild cognitive impairment: a cross-sectional study among the elderly in Chinese communities

**DOI:** 10.3389/fnagi.2022.1004954

**Published:** 2022-09-16

**Authors:** Guojun Liu, Yong Li, Yuzhen Xu, Wei Li

**Affiliations:** ^1^Department of Rehabilitation Medicine, The Third People’s Hospital of Lanzhou, Lanzhou, China; ^2^Department of Nephrology, Hubei Provincial Hospital of Traditional Chinese Medicine, Wuhan, China; ^3^Department of Nephrology, Affiliated Hospital of Hubei University of Chinese Medicine, Wuhan, China; ^4^Hubei Provincial Academy of Traditional Chinese Medicine, Wuhan, China; ^5^Department of Rehabilitation, The Second Affiliated Hospital of Shandong First Medical University, Taian, China; ^6^Department of Geriatric Psychiatry, Shanghai Mental Health Center, Shanghai Jiao Tong University School of Medicine, Shanghai, China; ^7^Alzheimer’s Disease and Related Disorders Center, Shanghai Jiao Tong University, Shanghai, China

**Keywords:** type 2 diabetes, cognition, elderly, MRI, the fourth ventricle

## Abstract

**Background:** Previous studies have confirmed that diabetes is associated with cognitive impairment, but there is little data on this among older Chinese.

**Methods:** This study included 192 dementia patients, 610 patients with mild cognitive impairment (MCI), and 2,218 normal controls. Their general demographic information (such as gender, age, education, etc.), disease-related information (hypertension), and diabetes information (such as whether you have diabetes, course of the disease, etc) were collected by standardized questionnaires. The mini-mental state examination (MMSE) and Montreal Cognitive Assessment (MoCA) were used to assess their overall cognitive function, Moreover, 84 healthy, randomly selected older adults also underwent brain MRI scans at the same time, and the target brain regions included the hippocampus, the third, fourth, and fifth ventricles.

**Results:** The proportion of type 2 diabetes was significantly higher in the dementia group (25.5%) than that in the normal elderly group (15.6%) and the MCI group (17.7%). By using stepwise multiple logistics regression analysis, we found that type 2 diabetes was associated with dementia (*p* = 0.005*, OR = 1.805, 95%CI: 1.199–2.761), but not with MCI (*p* > 0.05). The volume of the fourth ventricle of the healthy elderly with diabetes was significantly larger than that of the healthy elderly without diabetes (*p* < 0.05), but there was no statistical difference (*p* > 0.05) in the volume of the hippocampus, the third ventricle, and the fifth ventricle between the two groups. However, we did not find an association between the fourth ventricle and cognitive scores (MMSE and MoCA).

**Conclusions:** In conclusion, type 2 diabetes in elderly Chinese people is associated with dementia, but not MCI. Type 2 diabetes may impair cognitive function by affecting the volume of the fourth ventricle. However, larger longitudinal follow-up studies are needed to confirm these conclusions.

## Introduction

Dementia is a serious neurodegenerative disease that increases in incidence with age (Reddy and Hashmi, 2020). A recent meta-analysis suggested that the pooled prevalence of all-cause dementia among individuals aged 50 and over in the community was 697 (CI95%: 546–864) per 10,000 persons, and the number approximately doubles every 5 years (Cao et al., 2020). China has 249.49 million people aged 60 and above, accounting for 17.9% of the total population (140 million), indicating a higher prevalence of dementia (Cao et al., 2020). There is no doubt that caring for these dementia patients will place a heavy burden on the public and healthcare systems (Jia et al., 2020a). To make matters worse, there are currently no effective strategies to treat dementia or delay cognitive decline.

Mild cognitive impairment (MCI) is considered a transitional stage between unimpaired cognitive function and dementia (Jia et al., 2020b). It is defined as objective cognitive impairment relative to a person’s age and is associated with cognitive symptoms in a person with basically normal functional activities but without dementia (Cooper et al., 2015). According to some epidemiological studies, the prevalence of MCI is about 6 percent in the general population, while rising to 20 percent in people 65 and older (Lopez et al., 2007; Sachdev et al., 2015). People with MCI generally have a higher risk of developing dementia, with about 46 percent developing dementia within 3 years, compared with 3 percent of the same age group (Tschanz et al., 2006). However, not all patients with MCI will necessarily develop dementia, and a significant proportion of patients with MCI will revert to normal status after appropriate intervention (Karssemeijer et al., 2017). Therefore, there is a consensus that cognitive intervention should be initiated at the MCI stage (Jia et al., 2020b).

Of all the risk factors for dementia and MCI, diabetes stands out. Epidemiological studies confirm that people with diabetes are at increased risk of developing dementia and MCI (Biessels et al., 2006; Koekkoek et al., 2015), suggests that diabetes may play an important role in the pathogenesis of cognitive decline. Current information from neuroimaging and neuropathologic studies suggests that elderly patients with diabetes have significant evidence of subclinical infarction and cerebral atrophy (Mayeda et al., 2015). More interestingly, patients with diabetes often exhibit pathologic changes similar to those seen in early Alzheimer’s disease (AD; Ninomiya, 2014), and the mechanism may involve immunity, inflammation, and insulin resistance (Li et al., 2015). However, similar studies in China are relatively scarce, and their conclusions are also inconsistent. For example, in Jia et al. (2020b) study, they found that diabetes was a risk factor for both dementia and MCI, while other studies have not reached the same conclusion (Li et al., 2016; Qin et al., 2020).

In the current study, we used data from a large community sample of older adults to specifically explore the relationship between diabetes, dementia, and MCI. Unlike other studies, we also added structural magnetic resonance data to a subset of healthy people. Our hypothesis is that diabetes has an impact on both dementia and MCI, and that patients with diabetes already have changes in brain structure even when there is no significant change in their cognitive function.

## Methods

### The study population

Data were obtained from the China Longitudinal Aging Study (CLAS), which has been described in detail in our previous studies (Xiao et al., 2013; Lin et al., 2019). A total of 3,020 community-based individuals aged 60 years or older [including 610 patients with mild cognitive impairment (MCI), 192 patients with dementia, and 2,218 normal controls] were included in the current study. All subjects need to meet the following conditions: (1) age 60 and older; (2) vision and hearing were normal; (3) without a combination of life-threatening diseases, such as myocardial infarction, cerebral infarction, etc.; and (4) without serious mental illness, such as schizophrenia, major depression, etc. If these subjects were: (1) younger than 60 years of age; (2) associated with type 1 diabetes; (3) associated with diseases that affect blood glucose metabolism, such as Cushing’s syndrome; (4) associated with diseases that affect cognitive functions, such as syphilis and vitamin B12 deficiency, would be excluded; and (5) hyperthyroidism, Cushing syndrome, primary hyperaldosteronism or other diseases that affect glucose metabolism of the elderly would also be excluded. Then all the eligible elderly were required to complete a general demographic survey, neuropsychological assessment, and clinical assessment. The diagnosis of MCI was according to Petersen’s criteria (Petersen, 2011), while the diagnosis of dementia was according to DSM IV. All the diagnostic and evaluation procedures were performed by experienced clinicians (Biedermann and Fleischhacker, 2016). Moreover, T1 phase Brain MRI scans were also performed on 84 older adults with normal cognitive function in a random lottery.

This study was approved by the Ethics Committee of Shanghai Mental Health Center, and informed consent was signed by patients or their families before the study began. The whole study was carried out in accordance with the principles of the Declaration of Helsinki.

### Diagnosis of type 2 diabetes

Participants were considered to have type 2 diabetes if their fasting serum glucose level was ≥7.0 mmol/L (126 mg/dl) or their 2-h value in an oral glucose tolerance test was ≥11.1 mmol/L. Moreover, self-reported physician’s diagnosis or treatment with oral hypoglycemic agents and/or insulin were also considered to have type 2 diabetes (Gao et al., 2015).

### Cognitive assessment

The Mini-Mental State Examination (MMSE; Folstein et al., 1975) and Montreal Cognitive Assessment (MoCA; Nasreddine et al., 2005) are the most commonly used assessment tools in the field of geriatric cognition. Both of them have a total score of 30, and both have good sensitivity and specificity. However, compared with MMSE, the MoCA scale has better sensitivity. Therefore, the former was mainly used to screen for dementia, while the latter was mainly used to screen for MCI (Ciesielska et al., 2016).

### T1 phase structure MRI

Baseline cranial MRI was performed on 84 elderly subjects with normal cognitive function. T1 structural images were obtained using the Magnetom Verio 3.0 T scanner (Siemens, Munich, Germany), and the parameters were as follows: TR = 2,300 ms, TE = 2.98 ms, flip angle 9°, slice thickness 1.2 mm, matrix size 240*256, field of view (FOV) 240*256 mm, and 17,614 slices. All sMRI data were processed using FreeSurfer V6.0 software Clinica (Brown et al., 2020), including spatial registration, cortical thickness estimation, cortical surface segmentation, extraction of subcortical structures, and inclusion of blocks to 46 global structures. Previous studies have shown that the hippocampus is most closely related to cognitive function, while diabetes is often related to the increase in ventricular volume (Sadykova and Nazhmutdinova, 2009; Opitz, 2014; Eichenbaum, 2017; Lisman et al., 2017; Boutouyrie et al., 2022). Therefore, our target brain regions mainly included the hippocampus, third ventricle, fourth ventricle, and fifth brain region.

### Covariates

We also collected general demographic information (age, sex, education level), lifestyle information (smoking, drinking, tea drinking, physical exercise hobbies), and disease-related information (hypertension) through standardized questionnaires. Among these variables, those that differed among the MCI group, the dementia group, and the normal control group were considered covariates.

## Statistical Methods

Continuous variables were expressed by Mean ± Standard Deviation (SD), while classified variables were expressed by frequency (%). Univariate ANOVA and Chi-square test were used to compare continuous and categorical variables among the MCI group, dementia group, and normal control group, respectively. Stepwise multivariate logistic regression analysis was used to explore the association between type 2 diabetes and cognitive impairment (Model 1 only contained type 2 diabetes; model 2 contained type 2 diabetes, age, sex, and education levels; model 3 contained type 2 diabetes, age, sex, education levels, smoker, tea drinker, hobby, physical exercise, and hypertension). Next, the ROC curve was used to explore the sensitivity and specificity of type 2 diabetes in predicting dementia. Then the independent sample *T*-test was used to detect differences in brain area volume between the diabetes group and the non-diabetes group. Then the correlation analysis was used to explore the correlation between type 2 diabetes and cognitive scores and cognitive brain regions (gender, age, and education are controlled). Two-tailed tests were used at a significance level of *p* < 0.05 for all analyzes. The data were analyzed using SPSS 22.0 (IBM Corporation, Armonk, NY, USA).

## Results

### General demographic data of the research object

Table 1 presents the general demographic data of the research object. The proportion of type 2 diabetes was significantly higher in the dementia group (25.5%) than that in the normal elderly group (15.6%) and the MCI group (17.7%), while there was no statistical difference between the latter two groups. In addition, there were also statistical differences in age (*p* < 0.001), education (*p* < 0.001), gender (*p* < 0.001), smoker (*p* = 0.009), tea drinker (*p* < 0.001), hobby (*p* < 0.001), hypertension (*p* = 0.006), physical exercise (*p* < 0.001), MMSE (*P* < 0.001), and MoCA (*p* < 0.001) among the three groups, so these variables would eventually be considered as covariables.

**Table 1:** Demography, lifestyle, type 2 diabetes, and cognitive function in the overall database of study participants.

**Variables**	**Dementia (*n* = 192)**	**MCI (*n* = 610)**	**Normal (*n* = 2,218)**	**F/X^2^**	***p***
Age, years	78.83 ± 7.54	73.86 ± 8.24	70.10 ± 7.53	145.76	<0.001*
Education, years	4.34 ± 4.77	5.67 ± 5.02	9.25 ± 5.72	138.34	<0.001*
Males, n (%)	71 (37.0)	233 (38.2)	1,074 (48.4)	26.347	<0.001*
Smoker, n (%)	43 (22.4)	147 (24.1)	650 (29.3)	9.46	0.009*
Drinker, n (%)	31 (16.1)	119 (19.5)	475 (21.4)	3.65	0.162
Tea drinker, n (%)	57 (29.7)	220 (36.1)	1,116 (50.3)	61.39	<0.001*
Hobby, n (%)	48 (25.0)	267 (43.8)	1,318 (59.4)	116.98	<0.001*
Diabetes, n (%)	49 (25.5)	108 (17.7)	346 (15.6)	13.136	<0.001*
Hypertension, n (%)	112 (58.3)	294 (48.2)	1,029 (46.4)	10.244	0.006*
Physical Exercise, n (%)	92 (47.9)	389 (63.8)	1,689 (76.1)	94.35	<0.001*
MMSE	13.97 ± 7.41	22.38 ± 5.73	26.80 ± 3.51	877.04	<0.001*
MoCA	9.10 ± 6.25	16.72 ± 6.15	22.79 ± 5.18	724.15	<0.001*

Note: *p < 0.05; MoCA, Montreal Cognitive Assessment; MMSE, Mini-mental State Examination; MCI, mild cognitive impairment.

### Stepwise logistic regression model was used to investigate the relationship between type 2 diabetes, dementia, and MCI

**Figure 1:**
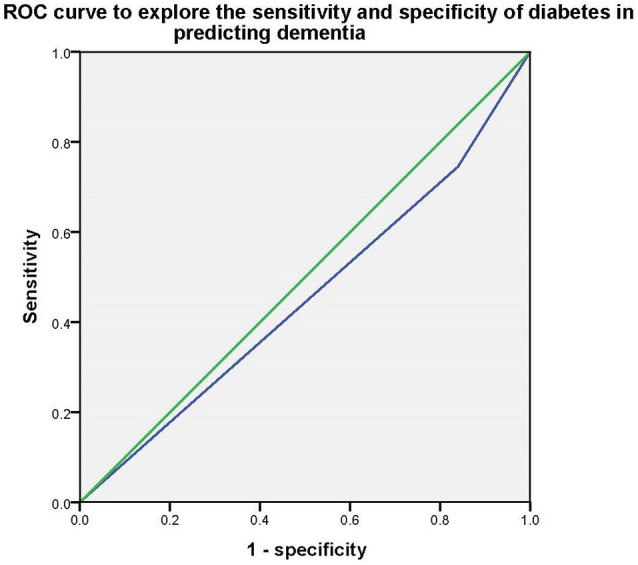
ROC curve to explore the sensitivity and specificity of diabetes in predicting dementia.

Stepwise multivariate logistic regression models were used to explore the association between type 2 diabetes and dementia/MCI. In model 1, without controlling for any variables, we found that type 2 diabetes was only associated with dementia (*p* < 0.001* OR = 1.854, 95%CI: 1.314–2.615), but not MCI (*p* > 0.05); In model 2, after controlling for age, education, and gender, we found that type 2 diabetes was also an influential factor for dementia (*p* = 0.003*, OR = 1.836, 95%CI: 1.237–2.724), but not MCI (*p* > 0.05); In model 3, smoker, tea drinker, hobbies, diabetes, and physical exercise were further controlled on the basis of Model 2, and the above association still existed (dementia: *p* = 0.005*, OR = 1.805, 95%CI: 1.199–2.761, MCI: *p* > 0.05). Then the Receiver Operating Characteristic (ROC) curve was used to investigate the sensitivity and specificity of type 2 diabetes in predicting dementia, and the area under the curve was 0.453 (*P* = 0.028, 95%CI: 0.409–0.497). Table 2 and Figure 1 presents the results.

**Table 2:** Different regression models were used to explore the association between diabetes and dementia.

**Variables**	**B**	**S.E**	**Wald**	**df**	***p***	**OR**	**95% confidence interval**
Dementia
Diabetes (model 1)	0.617	0.176	12.362	1	<0.001*	1.854	1.314–2.615
Diabetes (model 2)	0.607	0.201	9.101	1	0.003*	1.836	1.237–2.724
Age (model 2)	0.118	0.011	107.672	1	<0.001*	1.125	1.100–1.151
Education (model 2)	-0.117	0.019	38.090	1	<0.001*	0.889	0.857–0.923
Male (model 2)	0.171	0.183	0.876	1	0.349	1.187	0.829–1.699
Diabetes (model 3)	0.591	0.208	8.023	1	0.005*	1.805	1.199–2.761
Age (model 3)	0.106	0.012	84.782	1	<0.001*	1.112	1.087–1.138
Education (model 3)	-0.098	0.020	23.564	1	<0.001*	0.907	0.871–0.943
Male (model 3)	0.091	0.216	0.179	1	0.672	1.096	0.718–1.673
Smoker (model 3)	0.023	0.240	0.009	1	0.923	1.024	0.639–1.639
Tea (model 3)	-0.777	0.201	14.910	1	<0.001*	0.460	0.310–0.682
Physical exercise (model 3)	-0.691	0.177	15.202	1	<0.001*	0.501	0.345–0.709
Hobby (model 3)	-0.771	0.203	14.385	1	<0.001*	0.463	0.311–0.689
Hypertension (model 3)	0.322	0.177	3.332	1	0.068	1.380	0.977–1.951

Note: *p < 0.05.

### The association between type 2 diabetes and brain structure

**Figure 2:**
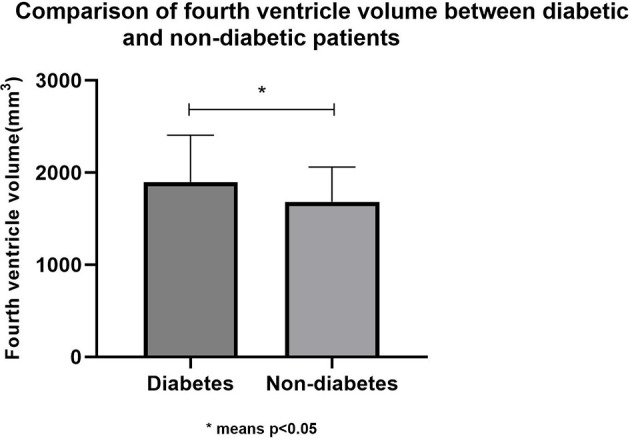
Comparison of fourth ventricle volume between diabetic and non-diabetic patients. *means *p* < 0.05.

To investigate the possible mechanism of type 2 diabetes affecting cognitive function, T1 structural cranial magnetic resonance imaging was performed on a random sample of healthy elderly people. Based on type 2 diabetes, 84 cognitively normal adults (their average age was 69.06 ± 6.66; there were 42 males and 42 females) were divided into two groups: diabetes (*n* = 36) and non-diabetes (*n* = 48). There were no statistically significant differences (*p* > 0.05) in age, education, sex, smoking, alcohol consumption, tea consumption, physical exercise, hobbies, hypertension, total brain volume, MMSE score, and MoCA score between the two groups. Therefore, in this study, the hippocampus volume and ventricular volume were mainly used as our main observation indicators. Finally, we found that the fourth ventricle volume of diabetic patients was significantly larger (*p* > 0.05) than that of non-diabetic patients, while there were no statistical differences in hippocampus, third ventricle volume, and fifth ventricle volume between the two groups. Table 3 and Figure 2 presents the results. However, we did not find any specific association between the fourth ventricle volume and cognitive score.

**Table 3:** Comparison of brain region structure and neuropsychological tests between diabetic and non-diabetic patients.

**Variables**	**Diabetes (*n* = 36)**	**Non-diabetes (*n* = 48)**	**X^2^ or t**	***p***
Age, years	68.50 ± 7.01	69.48 ± 6.43	-0.665	0.508
Education, years	9.18 ± 3.95	9.81 ± 4.15	-0.697	0.488
Male, n (%)	17 (47.2)	25 (52.1)	0.194	0.826
Smoker, n (%)	10 (27.8)	15 (31.3)	0.119	0.812
Drinker, n (%)	10 (27.8)	7 (14.6)	2.219	0.174
Tea drinker, n (%)	17 (47.2)	17 (35.4)	1.190	0.369
Take exercise, n (%)	22 (61.1)	30 (62.)	0.017	1.000
Hobby, n (%)	23 (63.9)	33 (68.8)	0.219	0.649
Hypertension, n (%)	29 (80.6)	45 (93.8)	3.415	0.091
Total brain volume, cm^3^	1,013.77 ± 104.35	1,012.08 ± 106.08	0.073	0.942
Left hippocampus, mm^3^	3,681.67 ± 417.06	3,710.50 ± 397.92	-0.322	0.748
Right hippocampus, mm^3^	3,925.16 ± 453.41	3,843.64 ± 454.51	0.814	0.418
3rd ventricle, mm^3^	1,546.58 ± 468.30	1,618.72 ± 622.81	-0.582	0.562
4th ventricle, mm^3^	1,898.39 ± 508.09	1,684.74 ± 376.02	2.216	0.029*
5th ventricle, mm^3^	0.00 ± 0.000	0.04 ± 0.202	-1.236	0.220
MMSE	27.50 ± 2.336	27.91 ± 2.244	-0.820	0.415
MoCA	23.50 ± 5.755	24.04 ± 3.759	-0.518	0.606

*p < 0.05. MoCA, Montreal Cognitive Assessment; MMSE, Mini-mental State Examination; MCI, mild cognitive impairment.

## Discussion

Both cognitive dysfunction and diabetes are common diseases among the elderly, and cognitive dysfunction is one of the important comorbidities of diabetes mellitus. There are different stages of diabetes-related cognitive dysfunction, each with different cognitive characteristics, affected age groups, and outcomes, which may also have different underlying mechanisms (Biessels and Despa, 2018). In the current study, we investigated the association between type 2 diabetes and cognitive dysfunction among the elderly in the Chinese community and found that: (1) type 2 diabetes was associated with dementia, but not MCI; and (2) older cognitively normal adults with type 2 diabetes had greater fourth ventricle volume compared with older cognitively normal adults without type 2 diabetes.

In our study, we found that dementia patients had a significantly higher rate of diabetes than normal elderly and MCI patients. Using stepwise logistic regression analysis, we found that type 2 diabetes was an important predictor of dementia, independent of gender, age, and education. However, no association was shown between type 2 diabetes and MCI. In Fei et al. (2013) study, they found that type 2 diabetes was associated with dementia and its subtypes amongst elderly people in the Chinese population. In Wang and Liu (2021) study, they found that early-onset diabetes had a stronger association with an increased risk of stroke, all dementia, and Alzheimer’s disease (AD) dementia than later-onset. In Shang et al. (2020) study, they found that diabetes, but not prediabetes, was associated with an increased risk of ischemic stroke and post-stroke dementia. In Jia L. et al.’s study, they found that type 2 diabetes was a risk factor for both dementia and MCI, but we did not find an association between type 2 diabetes and MCI. The reasons for the above differences may be as follows: (1) MCI was not divided into specific groups to distinguish between amnestic MCI and vascular MCI; (2) the sensitivity of males and females to diabetes was different; and (3) some confounding factors, such as APOE E4, may have a significant impact on the study results. Therefore, we are relatively certain that type 2 diabetes is closely associated with dementia, but the association with MCI needs to be further verified.

To investigate the possible mechanism of type 2 diabetes affecting the progression of dementia, we randomly selected some healthy elderly people with normal cognitive function and performed cranial magnetic resonance imaging of the T1 structural phase. Based on whether they had type 2 diabetes, those 84 cognitively normal elderly people were divided into diabetes (*n* = 36) and non-diabetes groups (*n* = 48), with no differences in gender, age, education, daily biochemical variables, disease-related variables, or cognitive scores. Although there was no difference in hippocampal volume or total brain volume between the two groups, the fourth ventricle volume was significantly larger in individuals with type 2 diabetes than in individuals without type 2 diabetes. Unfortunately, we did not find an association between the fourth ventricle and cognitive scores (MMSE and MoCA).

The fourth ventricle, also known as the cerebellum medulla cistern, is a closed chamber between the cerebellum and the medulla oblongata. The fourth ventricle is filled with cerebrospinal fluid. Its main function is buffering and nutritional support, and it is an important structure for cerebrospinal fluid to flow down and return to venous blood for reabsorption. Accumulated evidence shows that the fourth ventricle is closely related to blood glucose regulation, for example, in 1854, Claude Bernard, a French physiologist, discovered that a lesion at the base of the fourth ventricle in rabbits would cause sugar levels to rise in the blood (Tups et al., 2017). Moreover, it is now thought that the fourth ventricle may be an important medium for the brain to regulate blood sugar (Coppari, 2015). Since our sample size was small and no association between the fourth ventricle and cognitive score was found, we could not determine whether type 2 diabetes affected cognitive function by affecting the volume of the fourth ventricle. But the significance of our study is that it may provide new ideas for future research and new targets for finding brain structures in which glucose metabolism affects cognitive function.

We have to admit that there were some limitations in our study. Firstly, since this study was only a cross-sectional study, it could not indicate the causal effect between type 2 diabetes and cognitive impairment; secondly, information on diabetes was obtained through self-report rather than objective assessment, so there was the possibility of recall bias; thirdly, the sample size was relatively small, so we did not find an association between the fourth ventricle and cognitive function, so further expansion of the sample size is needed in the future; fourthly, we did not further distinguish between dementia and MCI subtypes, which may have influenced the results of the study.

## Conclusions

In conclusion, type 2 diabetes among the Chinese elderly is associated with a higher risk of dementia, but not MCI, and type 2 diabetes may impair cognitive function by affecting the volume of the fourth ventricle.

## Data Availability Statement

The raw data supporting the conclusions of this article will be made available by the authors, without undue reservation.

## Ethics Statement

The studies involving human participants were reviewed and approved by The Third People’s Hospital of Lanzhou. The patients/participants provided their written informed consent to participate in this study.

## Author Contributions

WL, YL, and GL contributed to the study concept and design. WL wrote this article. YX analyzed the data and drafted the manuscript. All authors contributed to the article and approved the submitted version.

## Funding

This study was supported by grants from the clinical research center project of Shanghai Mental Health Center (CRC2017ZD02), Shanghai Clinical Research Center for Mental Health (19MC1911100), the Cultivation of Multidisciplinary Interdisciplinary Project in Shanghai Jiaotong University (YG2019QNA10), curriculum reform of Medical College of Shanghai Jiaotong University, the Feixiang Program of Shanghai Mental Health Center (2020-FX-03), the National Natural Science Foundation of China (82101564), Chinese Academy of Sciences (XDA12040101), Shanghai Clinical Research Center for Mental Health (SCRC-MH, 19MC1911100), the National Natural Science Foundation of China (82001123), the Shanghai Science and Technology Committee (20Y11906800), Shandong Medical and Health Technology Development Fund (202103070325), Shandong Province Traditional Chinese Medicine Science and Technology Project (M-2022216), and Traditional Chinese medicine for chronic kidney disease (Provincial Key Laboratory Scientific Research special-19).
